# Bidirectional associations of recreational sedentary screen time and 24-h behaviors: a dynamic cross-sectional multilevel model analysis

**DOI:** 10.1186/s44167-026-00096-0

**Published:** 2026-02-01

**Authors:** Kristina Hasanaj, Krista S. Leonard, Dorothy D. Sears, Fang Yu, Megan E. Petrov, Sarah K. Keadle, Matthew P. Buman

**Affiliations:** 1 Edson College of Nursing and Health Innovation, Arizona State University, 550 N. 3rd Street, Phoenix, AZ 85004 USA; 2 Northwestern University Feinberg School of Medicine, Chicago, IL USA; 3 College of Health Solutions, Arizona State University, Phoenix, AZ USA; 4 Department of Kinesiology and Public Health, California Polytechnic State University, San Luis Obispo, CA USA

**Keywords:** Screen time, Television watching, TV viewing, Sedentary behavior, Physical activity, Sleep

## Abstract

**Background:**

Recreational sedentary screen time (rSST) is the most prevalent form of discretionary sedentary behavior and is strongly linked to poor health outcomes. However, the relationship between time spent in rSST and other 24-h behaviors is not well understood. The purpose of this study was to examine between- and within-day associations between rSST and other 24-h behaviors that include other non-rSST sedentary time (other-SED), standing (STAND), light physical activity (LPA), moderate-to-vigorous physical activity (MVPA), and total sleep (SLEEP).

**Methods:**

Baseline data from participants randomized to the StandUPTV study, an intervention aimed to reduce rSST in adults, were included. All 24-h behaviors were assessed continuously for 7-days. The activPAL device was used to assess rSST, other-SED, STAND, LPA, and MPVA; SLEEP was assessed using a GENEactiv accelerometer. rSST was collected using Wi-Fi plugs to capture TV time and tablet app usage. A multilevel modelling approach was used to assess bidirectional associations between rSST (total, daytime, evening) and 24-h behaviors at the between-person (across persons) and within-person (across days) levels, adjusting for age, sex, chronotype, education level, and week versus weekend day. The results were scaled hourly for interpretation.

**Results:**

On average, 8.0 ± 1.6 days of continuous daily 24-h behavior data were included from 94 participants (age [*M* ± *SD*: 42.3 ± 11.5] years; 82% female; 78% White; BMI [*M* ± *SD*: 29.8 ± 7.8] kg/m^2^). Greater total rSST was significantly associated with less other-SED (between-person b =  − 45.0, SE = 4.4, *p* < 0.01; within-person b =  − 44.5, SE = 2.0, *p* < 0.01). Similar results were observed when examining both daytime and evening rSST with other-SED. Negative associations were also observed between other-SED, STAND, LPA, and MVPA with rSST variables. No significant associations were observed between rSST variables and SLEEP.

**Conclusions:**

This is the first known analysis of the bidirectional relationship between rSST and 24-h behaviors. The negative association between rSST and other-SED suggests that rSST may displace rather than contribute to more cumulative sedentary time. These findings advocate that contexts of sedentary behavior should be considered as distinct behavioral targets in intervention development. Future interventions targeting rSST reduction should also include strategies to reduce total sedentary time.

Clinical Trials Registration: NCT04464993.

**Supplementary Information:**

The online version contains supplementary material available at 10.1186/s44167-026-00096-0.

## Background

A typical 24-h day can be categorized into different types and intensities of physical behaviors, including light-intensity physical activity (LPA) or moderate-to-vigorous physical activity (MVPA), standing, sedentary time, and sleep, that differ in their associations with health outcomes [1, 2]. For example, high levels of sedentary time, low levels of MVPA, and short sleep duration are associated with an increased risk of all-cause mortality, cardiovascular disease, metabolic syndrome, type 2 diabetes (T2D), and certain cancers [1–10]. Replacing sedentary time with more physical activity, or compensating for excessive sitting with higher MVPA, is associated with significant reductions in all-cause mortality among less active adults [11, 12].

Given that total time is constrained, increased sedentary time is associated with less time spent in other 24-h behaviors, such as total physical activity, LPA, and sleep duration [13]. Indeed, observational evidence suggests that the reallocation of time from sedentary to other physical activities is associated with better health outcomes, including reduced risk for all-cause mortality [14] and cardiovascular disease and cancer mortality [15]. However, these studies typically focus on average displacement effects based on weekly data. The relationship between time-use behaviors is thought to be dynamic and reciprocal; for example, longer nighttime sleep duration may lead to less sedentary time and MVPA the next day [16]. These associations are inconsistent and may depend on individuals’ age and sex, the timing of assessment (weekday vs. weekend), the type of assessment (reported vs. device-based), and chronotype (i.e., individualized preference for timing of sleep and wake across 24 h) [17–23]. Additionally, while previous research has examined total sedentary time in relation to other 24-h behaviors [17–23], understanding the predominant discretionary sedentary behavior (recreational sedentary screen time) and the impacts on time spent in other 24-h behaviors is not well understood.

Recreational sedentary screen time (rSST) is defined as discretionary time (i.e., not for work or educational purposes) spent watching screens, such as television (TV), computers, smartphones, tablets, or inactive video games. rSST is the most prevalent discretionary sedentary behavior outside of work and sleep, consuming over 4.5 h/day among American adults [24–26]. rSST is also associated with greater health risks than other sedentary time contexts (e.g., work, transport) [12, 14, 15, 27–33]. Notably, engaging in MVPA at 2–3 times greater than the current physical activity guidelines recommendations [34, 35] (~ 60–75 min/day of walking) lowered but did not fully attenuate the increased mortality risk associated with 5 + h/day of rSST [14]. Given the increasing prevalence of rSST and its robust association with adverse health outcomes, understanding the influence of this specific and contemporary context of sedentary time on 24-h behaviors is critical for developing effective domain-specific interventions to reduce sedentary behavior and increase physical activity.

The relationship between time spent in rSST and 24-h behaviors, both between- and within-participants, is not well understood. Understanding these relationships is necessary to determine whether specific behavior-context considerations are needed for successful intervention development [2]. To our knowledge, no studies have examined the relationships between rSST and 24-h behaviors using device-based measures of both screen time and physical behavior. The purpose of this study was to investigate the bidirectional associations between rSST and 24-h behaviors among individuals who reported high levels of screen time using continuous data from device-based measures.

## Methods

### Study design and procedure

This secondary analysis utilized baseline data from the StandUPTV randomized trial. The protocol and primary outcomes are described elsewhere [36, 37]. The StandUPTV study was a full factorial trial that aimed to develop an optimized intervention to reduce rSST in adults by > 60 min/day at 16 weeks. Participants were recruited through social media, research matches, newsletters, and online advertisements. After providing informed consent, participants were mailed a technology kit that included smart home devices (i.e., Raspberry Pi network device and WeMo smart plugs for each primary TV used), a pre-loaded tablet with regularly used applications (app), and a Fitbit Charge 4 (Fitbit LLC, San Francisco, CA), which collectively allowed for continuous real-time sedentary screen time tracking. A staff-assisted setup was provided to ensure proper technology setup and configuration for rSST measurement. Sociodemographic and other REDCap-administered survey assessments, clinical measures, and device-based measurements (activPAL, GENEActiv) were completed at baseline, 8 weeks, and 16 weeks. This study used only baseline data.

The study protocol was reviewed and approved by the Arizona State University Institutional Review Board (IRB #00012109). All participants received a full explanation of study procedures and potential risks and provided informed consent prior to any data collection. This research was conducted in accordance with the Declaration of Helsinki and relevant U.S. federal regulations governing human subjects research.

#### Participants

Eligible participants were healthy adults aged 23–64 years [36]. Inclusion criteria included self-reporting > 3 h/day of rSST, physical activity during leisure [38] and occupational time [39] categorized as below moderate activity, use of an Apple (iOS6+) or Android (2.3+) smartphone or tablet, access to home broadband internet or unlimited data plan, ability to read and understand English, and willingness to be randomized to any StandUPTV study conditions.

### Measures

#### Recreational sedentary screen time (rSST)

This was assessed using an integrated measure of minute-level posture and recreational screen time. The protocol provides a detailed description of the methods used to assess rSST [36]. A brief overview is provided here.

- Recreational screen time: recreational screen time was defined as a discretionary activity with screens for enjoyment that included socialization and communicating (e.g., Instagram, Facebook), TV viewing, video games, or leisure computer use. Screen time for educational (e.g., school, training) and work-related activities was not included as rSST time. Three categories of screen time were identified: TV/video, social media, and video games. Screen time was directly assessed using Wi-Fi plugs to monitor the home television power state (WeMo Insight Smart Plug, Belkin International, Inc.; Playa Vista, CA) and tablet app usage (Samsung Galaxy, Samsung). Participants could also log or adjust bouts of screen time that occurred outside the home or on alternative devices (e.g., smartphones) through the StandUPTV app. Screen time bouts viewed by another household member could also be rejected on the app. Participants were asked to use the tablet for screen time, including typical smartphone activities, because smartphone data could not be collected directly. However, smartphone usage was captured via summary metrics from the participant's smartphone device.
- Sedentary time: participants were asked to wear the activPAL3c micro accelerometer (PAL Technologies Ltd, Glasgow, Scotland) continuously during assessment periods to capture sedentary posture (i.e., any waking behavior in a seated or reclining posture at low energy expenditure) [40]. The device attachment procedures were based on several large-scale intervention studies [15, 28, 41]. The devices were waterproofed with a medical-grade adhesive covering and attached to the midline of the thigh with breathable, hypoallergenic tape, allowing for continuous 24-h wear for 7 consecutive days without removal for bathing or other water-based activities [36].

The screen time and activPAL data were merged at the minute level to determine rSST. An observation was labeled rSST if both conditions were true: (1) behavior was identified as sedentary by the activPAL, and (2) recreational screen time was identified by either the TV or tablet. Additional rSST variables include daily evening rSST minutes, defined as the total minutes of rSST between 5 pm and sleep time. Daily daytime rSST minutes were calculated by subtracting evening rSST from total rSST minutes. rSST focused on waking hours only (non-sleep), which was determined using a REDCap-based daily log to identify sleep and wake times [36, 42].

#### Other sedentary time, standing, and physical activity

activPAL3c data were used to further categorize waking behavior (software version 8.12, CREA algorithm). Any sedentary minute without recreational screen time was categorized as other non-rSST sedentary time (other-SED). Active time was categorized as standing (STAND), LPA (stepping < 100 steps/minute), and MVPA (stepping ≥ 100 steps/minute) [43]. Cycling was summarized with MVPA. Wear periods were excluded if continuous sitting or standing exceeded 6 h (considered non-wear), days had < 10 h of valid waking wear time, and participants had < 4 valid days.

#### Sleep

The wrist-worn GENEActiv (initialized to collect at 40 Hz) accelerometer (ActivInsights, Kimbolton, UK) was worn continuously (24 h/day) for a minimum of 7 consecutive days. The GENEActiv device is validated for sleep measurement [44–47], and the daily log completed by participants was used to differentiate sleep and wake periods [42]. An open-source and validated algorithm (GGIR package, R-software) [47] was used to process device and daily log data to provide sleep measures, including sleep duration, efficiency, latency, and wakefulness after sleep onset [48–51]. Sleep duration is defined as the minutes accumulated within the main sleep window. GGIR signal processing includes autocalibration using local gravity as a reference [49], detections of sustained abnormal high values, non-wear detection, and the average magnitude of dynamic acceleration calculation, corrected for average gravity over 5 s epochs and reported in milligravitational units (mg). Data files were excluded if the post-calibration error was > 0.01 g (10 mg), fewer than 1 day of valid wear [48, 52], or wear data were not present for each 15-min period of the 24-h cycle. Non-wear detection is described elsewhere [49]. The default non-wear setting was used, including invalid data imputed by the average at similar time points on different days during the assessment week [49]. Sleep duration was calculated using automated sleep detection (HDCZA sleep detection algorithm) [51] for missing log wake/sleep times. Nights with an average sleep duration of ≥ 200 and ≤ 840 min were included. Available nightly sleep duration (SLEEP) data were merged with corresponding daily behaviors.

#### Daily 24-h behavior summary

The data detailed above were merged to create a daily 24-h behavior summary that included the following variables: total rSST, daytime rSST, evening rSST, other-SED, STAND, LPA, MVPA, and SLEEP (subsequent night). rSST and other-SED variables were determined based on the a priori definition of recreational screen time (excluding work/education). rSST minutes were labeled only when the sedentary observation co-occurred with a recreational screen time event captured by the TV (Wi-Fi plug) or tablet (StandUPTV app). Sedentary minutes without a concurrent recreational screen time event were classified as other-SED. A shifted dataset was also created to merge 24-h behaviors with the previous night’s sleep. Data from participants with ≥ 4 days of valid wear (≥ 10 h of waking wear from activPAL and screen time data) were included in the analysis.

#### Demographic variables

Demographic variables were assessed to characterize the sample and identify potential confounders, including age, race/ethnicity, sex, marital status, living arrangements, education, employment status, occupation, and household income.

#### Chronotype

The Morningness-Eveningness Questionnaire (MEQ) was used to assess chronotype [53, 54]. This 19-item validated questionnaire assesses sleep habits, fatigue, and individual differences to determine the extent to which individuals are more active and alert at certain times of the day. The responses determine preferences in sleep and waking times and the subjective ‘optimal’ time when individuals feel at their best. Based on scores, individuals are classified as evening type (E-type; ≤ 52), intermediate or day type (D-type; 53–64), or morning type (M-type; ≥ 65) [55].

#### Body mass index

Self-reported height and body weight were used to calculate body mass index (BMI). Height was self-reported in inches. Body weight in pounds was measured using the Tanita BF-679 scale (Tokyo, Japan) provided. All participants were asked to collect two body weight measurements, take a photo of the scale display for each measurement, and upload the photos to the REDCap Clinical Measures Log. The research staff reviewed the submitted photos and entered the values. The average weight was calculated as the mean of the provided weight values. Average weight and self-reported height were used to calculate BMI (kg/m^2^).

### Data analysis

Participants with ≥ 4 days of ≥ 10 h of valid waking wear time from the activPAL and rSST data, and ≥ 1 valid nightly sleep data were included in this analysis. Bidirectional associations were examined between daily assessments of rSST variables (total, daytime, evening) and daily assessments of 24-h behaviors (other-SED, STAND, LPA, MVPA), as well as between the aforementioned variables and SLEEP (subsequent and previous nights), using a multilevel model (MLM) approach. This secondary analysis used all randomized participants with valid device data. Therefore, an a priori sample-size calculation for the present observational associations was not conducted. However, the analytic dataset included repeated daily observations per participant, which improves precision for within-person estimations.

**Fig. 1:**
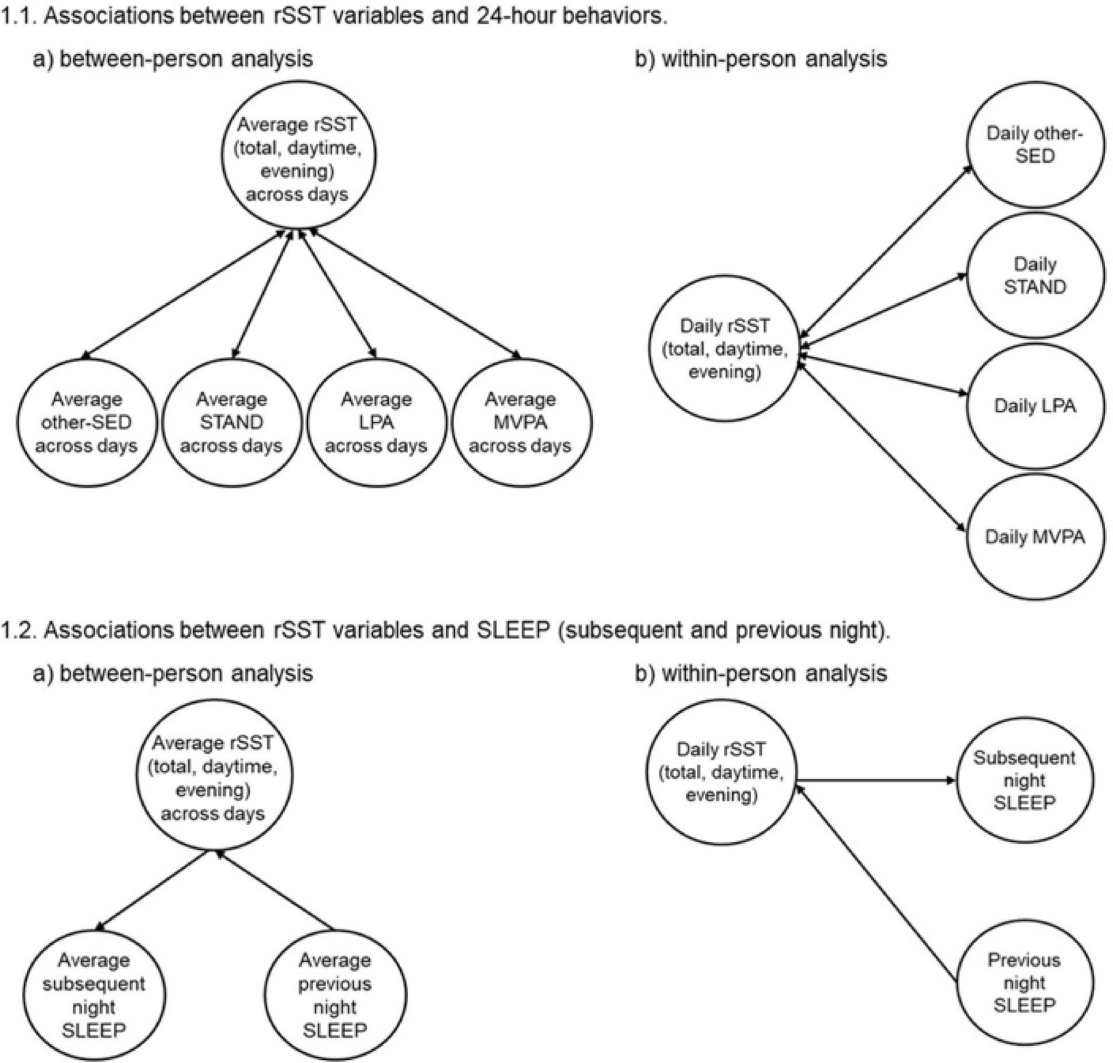
Multilevel models used to examine bidirectional associations between rSST and 24-h behaviors

The MLM approach can simultaneously examine the associations between rSST variables and 24-h behaviors at the between-person (across persons) and within-person (across days) levels. Between-person effects examine how person-level associations exist between 24-h behaviors (e.g., how an individual’s average total rSST is associated with average levels of 24-h behaviors). Within-person effects examine how daily associations exist between 24-h behaviors (e.g., how an individual’s daily departure from their typical total rSST is associated with daily 24-h behaviors), while controlling for the between-person associations. Separate models were examined for total, daytime, and evening rSST with each 24-h behavior, other-SED, STAND, LPA, MVPA, and SLEEP (subsequent and previous night). See Fig. 1 for a visual depiction of the models.

Demographic characteristics were included as covariates in the models to control for individual-level mean differences in the assessment of the dependent variables. Age (23–44 years vs. 45–64 years), BMI (normal (≥ 18.5 to < 25) vs. overweight (≥ 25 to < 30) or obese (≥ 30)), sex (female vs. male), chronotype (intermediate vs. morning and evening), day of the week (weekday vs. weekend day), and education (< bachelor’s degree vs. ≥ bachelor’s degree) were treated as categorical variables.

The statistical models were developed in two steps. Step 1 assessed the main effects associations between clinical and demographic covariates with rSST (total, daytime, evening), other-SED, STAND, LPA, MVPA, and SLEEP. The covariates include age, BMI, sex, chronotype, day of week, and education. Step 1 also covaries the fixed and random effects of intercept, so subsequent models control for individual trajectories of change in the dependent variable.

Step 2 then examined the bidirectional associations between rSST variables (total, daytime, evening) and 24-h behaviors. rSST variables were re-partitioned into between-person and within-person components by calculating person-level means (rSST variable [between-person]) and daily deviations from the person-level mean (rSST variable [within-person]), and then both parameters were simultaneously entered into the model. Variables were centered at the person-mean to address within-person variability. The same approach and models were applied to test associations between 24-h behaviors and rSST variables. For example, the beta coefficients, associated standard errors, and *p*-values can be interpreted as follows: (a) for total rSST (between-person): the association between average total rSST with the 24-h behavior of interest; and (b) for total rSST (within-person): the daily association between the deviation from the individual's typical total rSST with the 24-h behavior of interest.

Additional analyses were also conducted that examined the bidirectional interaction associations between rSST variables (total rSST, daytime rSST, evening rSST) and 24-h behaviors by the moderators of interest (age, sex, chronotype, and weekend/weekday) that are associated with daily behaviors. The beta coefficients, associated standard errors, and *p*-values for the interaction effects are interpreted as the magnitude by which the strength of the association between 24-h behavior and the dependent variable varies across levels of the putative moderator.

All results from Step 2 and the additional moderator analysis were scaled hourly to aid interpretability. Statistical significance was set at a *p*-value < 0.05. SAS Enterprise Guide, version 8.3 (SAS Institute, Inc., Cary, North Carolina), was used for analyses.

## Results

The StandUPTV study consented 177 participants, of whom 131 completed baseline measures, and 110 were randomized. The primary outcomes work provides more detail [37]. Only the 110 randomized participants were included in this secondary analysis, and the final analytic sample comprised of 94 participants with valid measures based on ≥ 4 days of ≥ 10 h of valid waking wear time from the activPAL and rSST data, and ≥ 1 valid nightly sleep data. Table 1 presents participants' clinical and demographic characteristics. The sample had a mean age of 42.3 ± 11.5 years, BMI: 29.8 ± 7.8 kg/m^2^, and were 81.9% female. The majority of the sample were adults between the ages of 23–44 years (62.8%), non-Hispanic Whites (77.7%), with an intermediate chronotype (50%), and who earned a bachelor’s degree or higher (76.6%). The means and standard deviation for 24-h behaviors are presented in Table 2. Additional participant characteristics are provided in Supplemental Table 1 (see Additional File 1), and the main effects of clinical and demographic covariates on 24-h behaviors are presented in Supplemental Table 2 (see Additional File 1).

**Table 1:** Participant clinical and demographic characteristics

	Total (*n* = 94)
Age (years)	42.3 (11.5)
23–44 years	59 (62.8)
45–64 years	35 (37.2)
Sex (female)	77 (81.9)
Body mass index (kg/m^2^)	29.8 (7.8)
Normal	27 (28.7)
Overweight	32 (34.0)
Obese	35 (37.2)
Ethnicity	
Hispanic/Latino	21 (22.3)
Race	
White	73 (77.7)
Black or African American	5 (5.3)
Asian	9 (9.6)
Native American or Alaskan Native	2 (2.1)
Pacific Islander	1 (1.1)
Other	6 (6.4)
Education	
Obtained GED	1 (1.1)
High school graduate	1 (1.1)
Completed some college credit, no degree	14 (14.9)
Associate’s degree	6 (6.4)
Bachelor’s degree	29 (30.9)
Master’s, professional, or doctoral degree	43 (45.7)
Chronotype	
Evening	8 (8.5)
Morning	39 (41.5)
Intermediate	47 (50.0)

Data are n (%); age and body mass index are mean (SD)

**Table 2:** Daily means and standard deviations for rSST and 24-h behaviors

	Total (*n* = 94)
Waking day (activPAL) (min)	
Total sedentary time	638.8 (96.1)
Total rSST	182.5 (125.7)
Daytime rSST	81.6 (80.3)
Evening rSST	101.0 (60.0)
Other non-rSST sedentary time	456.3 (138.8)
Standing time	221.3 (68.5)
Light physical activity	68.7 (28.1)
Moderate-vigorous physical activity	18.6 (15.6)
Device wear time	947.5 (50.1)
Device non-wear time	18.5 (21.9)
Sleep (GENEActiv)	(*n* = 86)
Time in bed (min)	470.7 (51.8)
Sleep duration (min)	410.2 (49.1)
Nights (count)	7.2 (1.8)
Non-wear (%)	0.3 (0.9)
Daily summary	
24-h day (wake and sleep) (min)	1361.7 (45.8)
Total waking day (min)	947.5 (50.1)
Daytime (min)	588.3 (79.0)
Evening (min)	359.6 (75.5)
Steps (count)	6780.8 (2851.2)
Included days (count)	8.0 (1.6)

The data are mean (SD) from participants with valid daily activPAL (*n* = 94) data (≥ 4 days of ≥ 10 waking hours and available screen time) and nightly GENEActiv data (*n* = 86). rSST = recreational sedentary screen time

### Total rSST and 24-h behaviors

#### Associations between total rSST with 24-h behaviors

The associations revealed that at the between-person level, each 1-h increase in average total rSST significantly displaced other-SED by 46 min and STAND by 10 min, but was not significantly associated with LPA and MVPA (Table 3). The within-person level associations indicated that each 1-h increase of an individual’s average total rSST significantly displaced other-SED by 43 min, STAND by 9 min, LPA by 2 min, and MVPA by 1 min.

**Table 3:** Multilevel models of the relationships between total rSST and 24-h behaviors

	Model 1a: total rSST → other-SED			Model 1b: total rSST → STAND			Model 1c: total rSST → LPA			Model 1d: total rSST → MVPA		
Parameter	β	SE	*p*	β	SE	*p*	β	SE	*p*	β	SE	*p*
Total rSST (between-person)	** − 45.73**	**4.26**	** < .01**	** − 9.77**	**3.11**	** < .01**	− 1.73	1.39	0.21	− 0.83	0.76	0.28
Total rSST (within-person)	** − 43.27**	**1.92**	** < .01**	** − 9.38**	**1.43**	** < .01**	** − 2.09**	**0.56**	** < .01**	** − 0.70**	**0.27**	**0.01**

	Model 2a: other-SED → total rSST			Model 2b: STAND → total rSST			Model 2c: LPA → total rSST			Model 2d: MVPA → total rSST		
Parameter	β	SE	*p*	β	SE	*p*	β	SE	*p*	β	SE	*p*
24-h behavior (between-person)	** − 43.71**	**4.03**	** < .01**	** − 34.41**	**11.18**	** < .01**	− 33.07	27.53	0.23	− 55.82	50.24	0.27
24-h behavior (within-person)	** − 36.09**	**1.61**	** < .01**	** − 23.68**	**3.59**	** < .01**	** − 35.89**	**9.55**	** < .01**	** − 50.11**	**19.64**	**0.01**

Beta estimates (β) and standard errors (SE) are scaled hourly. Significant *p*-values (*p*) are bolded (*p* < 0.05). Data are in minutes

Model 1 examined the associations of total rSST (independent variable) with 24-h behaviors, other-SED (model 1a), STAND (model 1b), LPA (model 1c), and MVPA (model 1d) (dependent variables)

Model 2 examined the associations of 24-h behaviors, other-SED (model 2a), STAND (model 2b), LPA (model 2c), and MVPA (model 2d), (independent variables) with total rSST (dependent variable)

All models were adjusted for age, sex, chronotype, BMI, weekday/weekend, education, and the linear associations of change in the outcome

rSST, Recreational sedentary screen time; other-SED, Other non-rSST sedentary time; STAND, Standing time; LPA, Light-physical activity; MVPA, Moderate-vigorous physical activity

#### Associations between total rSST with subsequent night SLEEP

The results indicate that each 1-h increase in average total rSST was associated with a displacement of the subsequent night’s sleep by 3 min at the between-person level (Table 5). At the within-person level, each 1-h increase in an individual’s average total rSST was associated with an increase in the subsequent night’s SLEEP by 1 min. However, the results were not statistically significant.

#### Associations between 24-h behaviors with total rSST

The analysis revealed that at the between-person level, each 1-h increase in average other-SED and STAND significantly displaced total rSST by 44 and 34 min, respectively, but was not significantly associated with LPA and MVPA (Table 3). The within-person level associations indicated that each 1-h increase of an individual’s average other-SED, STAND, LPA, and MVPA significantly displaced total rSST by 36, 24, 36, and 50 min, respectively.

#### Associations between previous night SLEEP with next day total rSST

The results show that each 1-h increase in the previous night’s SLEEP wasn’t significantly associated with the next day’s rSST at the between- or within-person level (Table 5).

### Daytime rSST and 24-h behaviors

The between-person analysis of daytime rSST and 24-h behaviors shows that each 1-h increase in average daytime rSST significantly displaced other-SED by 66 min and STAND by 15 min (Table 4). At the within-person level, each 1-h increase of an individual’s average daytime rSST significantly displaced other-SED by 56 min, STAND by 12 min, LPA by 3 min, and MVPA by 1 min.

**Table 4:** Multilevel models of the relationships between daytime and evening rSST and 24-h behaviors

	Model 1.1a: daytime rSST → other-SED			Model 1.1b: daytime rSST → STAND			Model 1.1c: daytime rSST → LPA			Model 1.1d: daytime rSST → MVPA		
Parameter	β	SE	*p*	β	SE	*p*	β	SE	*p*	β	SE	*p*
Daytime rSST (between-person)	** − 66.35**	**7.37**	** < .01**	** − 15.18**	**4.93**	** < .01**	− 3.36	2.18	0.12	− 1.27	1.20	0.29
Daytime rSST (within-person)	** − 55.57**	**3.09**	** < .01**	** − 11.74**	**2.12**	** < .01**	** − 2.68**	**0.83**	** < .01**	** − 1.25**	**0.40**	** < .01**

	Model 1.2a: other-SED → daytime rSST			Model 1.2b: STAND → daytime rSST			Model 1.2c: LPA → daytime rSST			Model 1.2d: MVPA → daytime rSST		
Parameter	β	SE	*p*	β	SE	*p*	β	SE	*p*	β	SE	*p*
24-h behavior (between-person)	** − 25.36**	**2.78**	** < .01**	** − 21.40**	**7.10**	** < .01**	− 26.15	17.33	0.13	− 34.20	31.76	0.28
24-h behavior (within-person)	** − 21.31**	**1.19**	** < .01**	** − 13.64**	**2.46**	** < .01**	** − 21.07**	**6.49**	** < .01**	** − 41.05**	**13.29**	** < .01**

	Model 2.1a: evening rSST → other-SED			Model 2.1b: evening rSST → STAND			Model 2.1c: evening rSST → LPA			Model 2.1d: evening rSST → MVPA		
Parameter	β	SE	*p*	β	SE	*p*	β	SE	*p*	β	SE	*p*
Evening rSST (between-person)	** − 84.61**	**10.16**	** < .01**	** − 16.28**	**6.66**	**0.01**	− 1.67	2.93	0.57	− 1.40	1.60	0.38
Evening rSST (within-person)	** − 54.87**	**3.95**	** < .01**	** − 12.34**	**2.55**	** < .01**	** − 2.68**	**0.99**	**0.01**	− 0.40	0.49	0.42

	Model 2.2a: other- SED → evening rSST			Model 2.2b: STAND → evening rSST			Model 2.2c: LPA → evening rSST			Model 2.2d: MVPA → evening rSST		
Parameter	β	SE	*p*	β	SE	*p*	β	SE	*p*	β	SE	*p*
24-h behavior (between-person)	** − 18.43**	**2.18**	** < .01**	** − 12.99**	**5.46**	**0.02**	− 6.82	13.23	0.61	− 21.77	24.05	0.37
24-h behavior (within-person)	** − 14.75**	**1.07**	** < .01**	** − 10.05**	**2.07**	** < .01**	** − 14.85**	**5.45**	**0.01**	− 9.03	11.21	0.42

Beta estimates (β) and standard errors (SE) are scaled hourly. Significant *p*-values (*p*) are bolded (*p* < 0.05). Data are in minutes

Model 1.1 examined the associations of daytime rSST (independent variable) with 24-h behaviors, other-SED (model 1.1a), STAND (model 1.1b), LPA (model 1.1c), and MVPA (model 1.1d) (dependent variables)

Model 1.2 examined the associations of 24-h behaviors, other-SED (model 1.2a), STAND (model 1.2b), LPA (model 1.2c), and MVPA (model 1.2d), (independent variables) with daytime rSST (dependent variable)

Model 2.1 examined the associations of evening rSST (independent variable) with 24-h behaviors, other-SED (model 2.1a), STAND (model 2.1b), LPA (model 2.1c), and MVPA (model 2.1d) (dependent variables)

Model 2.2 examined the associations of 24-h behaviors, other-SED (model 2.2a), STAND (model 2.2b), LPA (model 2.2c), and MVPA (model 2.2d), (independent variables) with evening rSST (dependent variable)

All models were adjusted for age, sex, chronotype, BMI, weekday/weekend, education, and the linear associations of change in the outcome

rSST, Recreational sedentary screen time; other-SED, Other non-rSST sedentary time; STAND, Standing time; LPA, Light-physical activity; MVPA, Moderate-vigorous physical activity

The analysis between 24-h behaviors with daytime rSST revealed that at the between-person level, each 1-h increase in average other-SED and STAND significantly displaced daytime rSST by 25 min and 21 min, respectively (Table 4). Associations with LPA and MVPA were not significant. The within-person level associations indicated that each 1-h increase of an individual’s average other-SED, STAND, LPA, and MVPA significantly displaced daytime rSST by 21, 14, 21, and 41 min, respectively. Daytime rSST was not significantly associated with the previous or subsequent night’s SLEEP (Table 5).

**Table 5:** Multilevel models of the associations between rSST variables and SLEEP

	Total rSST			Daytime rSST			Evening rSST		
	Model 3a: total rSST → subsequent night SLEEP			Model 3b: daytime rSST → subsequent night SLEEP			Model 3c: evening rSST → subsequent night SLEEP		
Parameter	β	SE	*p*	β	SE	*p*	β	SE	*p*
rSST variable (between-person)	− 2.7	2.06	0.19	− 4.97	3.23	0.12	− 3.32	4.47	0.46
rSST variable (within-person)	0.60	1.36	0.66	2.78	1.93	0.15	− 2.56	2.48	0.30

	Total rSST			Daytime rSST			Evening rSST		
	Model 4a: previous night SLEEP → next day total rSST			Model 4b: previous night SLEEP → next day daytime rSST			Model 4c: previous night SLEEP → next day evening rSST		
Parameter	β	SE	*p*	β	SE	*p*	β	SE	*p*
SLEEP (between-person)	− 26.44	18.73	0.16	− 18.38	12.02	0.13	− 8.23	8.84	0.35
SLEEP (within-person)	− 0.89	5.12	0.86	1.13	3.55	0.75	− 2.03	2.86	0.48

Beta estimates (β) and standard errors (SE) are scaled hourly. Significant *p*-values (*p*) are bolded (*p* < 0.05). Data are in minutes

Model 3 examined the association of total rSST (3a), daytime rSST(3b), and evening rSST (3c) variables (independent variables) with the subsequent night SLEEP (dependent variable)

Model 4 examined the association of the previous night’s SLEEP (independent variable) with the next day’s total rSST (4a), daytime rSST (4b), and evening rSST (4c) variables (dependent variables)

All models were adjusted for age, sex, chronotype, BMI, weekday/weekend, education, and the linear associations of change in the outcome

rSST, Recreational sedentary screen time; SLEEP, Sleep duration

### Evening rSST and 24-h behaviors

The associations between evening rSST with 24-h behaviors revealed that at the between-person level, each 1-h increase in average evening rSST significantly displaced other-SED by 85 min and STAND by 16 min (Table 4). The within-person level associations indicated that each 1-h increase of an individual’s average evening rSST significantly displaced other-SED by 55 min, STAND by 12 min, and LPA by 3 min.

The analysis between 24-h behaviors with evening rSST revealed that at the between-person level, each 1-h increase in average other-SED and STAND significantly displaced evening rSST by 18 min and 13 min, respectively (Table 4). The within-person level associations indicated that each 1-h increase of an individual’s average other-SED, STAND, and LPA significantly displaced evening rSST by 15, 10, and 15 min, respectively. Increased evening rSST was not associated with the subsequent night's sleep or the next day’s SLEEP (Table 5).

### Interaction associations between RSST variables and 24-h behaviors

The interaction results between rSST variables and 24-h behaviors by moderators (age, sex, chronotype, and weekend/weekday) can be found in Supplemental Tables 3–6 (see Additional File 1). For interaction associations between total rSST and 24-h behaviors, significant negative associations were observed between total rSST and LPA by age (between-person) and by morning chronotype (within-person). Considering daytime rSST and 24-h behaviors, the interaction associations showed significant negative associations between daytime rSST with other-SED by evening chronotype (within-person), and with LPA by age (between-person), morning chronotype (within-person), and weekend day (within-person). The interaction associations between evening rSST with 24-h behaviors resulted in significant positive associations between evening rSST with LPA by sex (within-person) and with subsequent night SLEEP by sex (within-person). See Supplemental Tables 3–6 (Additional File 1).

## Discussion

This study examined the bidirectional associations between rSST (total, daytime, and evening) and 24-h behaviors (other-SED, STAND, LPA, MVPA, SLEEP) using continuous device-based measures. Overall, greater total rSST was significantly associated with less other-SED and had small, statistically significant associations with STAND, LPA, and MVPA. Negative associations were also observed between other-SED, STAND, LPA, and MVPA with total rSST. Results for daytime and evening rSST and 24-h behaviors showed associations similar to those for total rSST. No significant associations were observed between rSST variables and SLEEP.

Our results are comparable to previous studies reporting negative associations between rSST and physical activity, yielding very modest associations with MVPA [56, 57]. Prior studies have used a combination of self-report, mobile applications, and wearable devices to measure rSST and have reported overall positive associations between rSST and other-SED [60, 61, 69]. However, the results from this study revealed bidirectional negative associations between rSST variables and other-SED at both between- and within-person levels. Suggesting that increases in rSST variables are associated with lower other-SED and vice versa. For total rSST, the between- (b =  − 45.7 min) and within-person (b =  − 43.3 min) level associations with other-SED are comparable. Whereas, compared to total rSST, the associations between daytime rSST with other-SED are larger at the between- and within-person levels. However, the associations between evening rSST with other-SED are larger compared to both total and daytime rSST at the between- and within-person levels. This suggests, for example, that each hour of evening rSST is associated with an 85-min displacement of other-SED. These findings indicate a negative relationship between rSST and other-SED, which contrasts with the current literature suggesting a positive relationship. This discrepancy may be due to improved measurement approaches and consideration of the 24-h day. Prior research studies [57, 60, 61] have relied on self-report measures of rSST, with few studies including mobile applications to capture smartphone use [62–65] or television sounds [59] as proxies to assess screen time. Similar approaches that incorporate wearable devices have also been used to measure other sedentary time, physical activity, and sleep [56, 66, 67]. Several studies have assessed smartphone use, TV time, and their combination, along with other screen time contexts; however, multiple behavioral domains were not examined [62–65].

Rouse and Biddle [68] assessed specific sedentary behavior contexts among university students and found a negative association between technology (TV, video games, computer)- and study-based sedentary time. Whereas Wagnild and Pollard [60] compared the activPAL-measured sedentary time and TV time in a sample of pregnant women and found no significant associations between TV time and total sedentary time. However, they reported significant associations between high TV time (vs. low TV time) and high evening sedentary time during the evening hours (6–11 pm) among women [60]. Several studies [58, 60, 61, 69] have suggested that greater rSST is associated with increased sedentary time, which can lead to poor health outcomes. However, the results from this study highlight a different relationship, suggesting that not only could rSST be displacing more active behaviors, but also displacing other forms of sedentary time, such as reading and spending time with family and friends.

Prior literature has assessed the bidirectional relationships between sleep and rSST, where adults with an evening chronotype, short sleep duration, and insomnia symptoms had higher odds of high discretionary screen time at follow-up [70]. Other research studies have posited a negative association between rSST and sleep duration. Sampasa-Kanyinga et al. [70] found that evening chronotype, short sleep duration, and insomnia symptoms were associated with greater discretionary screen time in a large population-based prospective cohort. Other studies have reported an association between greater discretionary sedentary screen time and shorter sleep duration, and we did not observe significant associations in the present study [64, 71–73]. We enrolled a sample of individuals motivated to reduce rSST, and there may not have been sufficient variance in rSST. More work is needed to better understand these relationships.

The notable strengths of this study are the novel use of continuous, device-based measures to assess rSST and 24-h behaviors across consecutive days, and the inclusion of specific behavior contexts of total, daytime, and evening rSST. This work provides a new perspective on the associations between rSST and other-SED, as well as other 24-h behaviors. The implications of this work improve understanding of the relationships between device-based measures of rSST and 24-h behaviors in sedentary adults, supporting future intervention development to optimize interventions that address multiple health behaviors.

However, this work is not without limitations. The sample was predominantly non-Hispanic White, highly educated, inactive females who reported high screen time. Participants were encouraged to use the tablet for all recreational screen time, but we could not directly assess smartphone screen time. Participants were encouraged to use the study tablet for rSST to support standardized measurement and log any additional screen time outside the home or via other devices (i.e., smartphones). Still, smartphone-based rSST may be underrepresented in our analysis because smartphone data could not be collected directly. Some misclassification of rSST is possible because TV power state/tablet usage may not perfectly reflect actual viewing or recreational engagement. We mitigated this by defining rSST only when sedentary posture and a concurrent TV/tablet screen-time event co-occurred, and by allowing participants to reject/adjust screen-time bouts in the app. Assessing the relationship between long and short bouts of rSST and other-SED would provide further insight into these sedentary behavior contexts and their associations with other 24-h behaviors. Beyond rSST, device-based assessment of other sedentary contexts was not measured. Work schedules (e.g., work hours, shift timing) likely structure opportunities for rSST and other 24-h behaviors. Future studies should incorporate detailed work-hour measures to evaluate whether associations differ by occupational time constraints. Additionally, rSST context (i.e., social media, smartphones, video games) can contribute to displacement patterns across the day and potentially influence the relationships between behaviors, but was not assessed in this work. Future studies should assess whether social-media rSST differs from TV- or gaming-related rSST in its bidirectional associations with 24-h behaviors.

This work focused on sleep duration in the present analyses, and future work should examine whether rSST timing and context are more strongly associated with sleep quality and sleep continuity than with sleep duration alone, to further elucidate these relationships. In addition, sleep estimates may be less reliable for participants contributing fewer valid nights of sleep data. This work required only one valid night to maximize sample retention, which may increase measurement error and attenuate associations. Prior reliability work indicates that single-night estimates have limited reliability and that multiple nights are required to estimate habitual sleep duration, particularly across weekdays versus weekends [74]. However, only one participant contributed 1 night of data, and 82/86 (95%) of the sample contributed 6 + valid nights for this analysis, and we did not require a higher minimum to avoid excluding otherwise valid behavior days. Overall, this work offers a new perspective on the associations between rSST and 24-h behaviors and underscores the need for further research.

## Conclusions

These results provide a comprehensive perspective on rSST and 24-h behaviors and present evidence of the bidirectional relationship between rSST and other-SED, and a modest relationship with physical activity. This work used device-based measures to assess continuous rSST and 24-h behaviors data across consecutive days. Negative associations were observed between rSST variables and other-SED, suggesting that rSST may displace sedentary time rather than contribute to additional total sedentary time. Likely reflecting a shift in sedentary behavior context composition rather than a simple reduction in overall sedentary time. The implications of this work suggest that total and specific contexts of sedentary behavior (i.e., total sedentary time and rSST) should be considered individually as behavioral targets in intervention development. This work also highlights that rSST constitutes as a distinct sedentary domain that may require intervention strategies beyond reducing sedentary time broadly. Future interventions targeting rSST reduction to increase physical activity should also include strategies designed to reduce total sedentary time. More work is needed using device-based measures to understand the influence of rSST on 24-h behaviors, to further characterize these behavioral relationships across the 24-h day, and to assess their impact on health outcomes and inform future public health recommendations for behavior change.

## Supplementary Information

Below is the link to the electronic supplementary material.

Supplementary Material 1

Supplementary Material 2

## Data Availability

The datasets used for the current study are available from the corresponding author upon reasonable request.

## Abbreviations

rSST: Recreational sedentary screen time
other-SED: Other non-rSST sedentary time
STAND: Standing
LPA: Light-intensity physical activity
MVPA: Moderate-to-vigorous physical activity
SLEEP: Sleep duration
TV: Television viewing
REDCap: Research electronic data capture
MEQ: Morningness-Eveningness Questionnaire
E-type: Evening chronotype
D-type: Intermediate (or day) chronotype
M-type: Morning chronotype
kg/m^2^: Kilograms/meters^2^ (BMI unit)
Wi-Fi: Wireless fidelity
Hz: Hertz
GGIR: Generation Generic Interface for Research; open-source R-package for sleep data processing
mg: Milligravitational units
HDCZA: The algorithm used in GGIR when the sleep log is not present
MLM: Multilevel modeling
SAS: Statistical analysis system program
