# Supplementary material for: Bidirectional associations of recreational sedentary screen time and 24-h behaviors: a dynamic cross-sectional multilevel model analysis
Source: J Act Sedentary Sleep Behav. 2026 Feb 1;5:4. doi: 10.1186/s44167-026-00096-0 (PMC12961888; doi:10.1186/s44167-026-00096-0)
Supplement: Supplementary file 1 — Supplementary Material 1 [file 44167_2026_96_MOESM1_ESM.docx]

| **Supplemental Table 1.** Additional participant demographic characteristics**.** | | | |
| --- | --- | --- | --- |
|  | **Total (n=94)** | | |
|  | **n** |  | **%** |
| Marital Status |  |  |  |
| Single, never married | 25 |  | 26.6 |
| Married or partnered | 54 |  | 57.5 |
| Separated/divorced | 10 |  | 10.6 |
| Widowed | 5 |  | 5.3 |
| Living Arrangements |  |  |  |
| Parents' Home | 4 |  | 4.3 |
| Rent or share rent | 31 |  | 33.0 |
| Own a home, making mortgage payments | 45 |  | 47.9 |
| Own a home, home fully paid off | 12 |  | 12.8 |
| Other | 2 |  | 2.1 |
| People Living in Household |  |  |  |
| 0 people one | 11 |  | 11.7 |
| 1-3 people one | 69 |  | 73.4 |
| 4-6 people one | 14 |  | 14.9 |
| Work Status |  |  |  |
| Going to school | 17 |  | 18.1 |
| Working full-time | 66 |  | 70.2 |
| Working part-time | 14 |  | 14.9 |
| Working in the home/homemaker | 9 |  | 9.6 |
| Retired | 1 |  | 1.1 |
| Unemployed | 4 |  | 4.3 |
| Other | 1 |  | 1.1 |
| Occupation |  |  |  |
| Executive, administrator, or senior manager | 5 |  | 5.3 |
| Professional | 36 |  | 38.3 |
| Technical support | 4 |  | 4.3 |
| Sales | 1 |  | 1.1 |
| Clerical and administrative support | 22 |  | 23.4 |
| Service, Hospitality, and Food | 3 |  | 3.2 |
| Student | 10 |  | 10.6 |
| Unemployed | 7 |  | 7.5 |
| Other | 6 |  | 6.4 |
| Household Income |  |  |  |
| $14,999 or less | 5 |  | 5.3 |
| $15,000 - $24,999 | 1 |  | 1.1 |
| $25,000 - $34,999 | 2 |  | 2.1 |
| $35,000 - $49,999 | 6 |  | 6.4 |
| $50,000 - $74,999 | 19 |  | 20.2 |
| $75,000 - $99,999 | 16 |  | 17.0 |
| $100,000 - $124,999 | 22 |  | 23.4 |
| $125,000 - $149,999 | 9 |  | 9.6 |
| $150,000 - $174,999 | 2 |  | 2.1 |
| $175,000 - $199,999 | 2 |  | 2.1 |
| $200,000 - $249,999 | 4 |  | 4.3 |
| $250,000 - $299,999 | 1 |  | 1.1 |
| Other | 5 |  | 5.3 |

| **Supplemental Table 2**. Clinical and demographic covariate associations between rSST and 24-hour behaviors. | | | | | | | | | | | | | | | | | | | | | | | | |
| --- | --- | --- | --- | --- | --- | --- | --- | --- | --- | --- | --- | --- | --- | --- | --- | --- | --- | --- | --- | --- | --- | --- | --- | --- |
|  | Total rSST | | | Daytime rSST | | | Evening rSST | | | other-SED | | | STAND | | | LPA | | | MVPA | | | SLEEP | | |
| Parameter | β | SE | p | β | SE | p | β | SE | p | β | SE | p | β | SE | p | β | SE | p | β | SE | p | β | SE | p |
|  |  |  |  |  |  |  |  |  |  |  |  |  |  |  |  |  |  |  |  |  |  |  |  |  |
| Age (ref 23-44 yrs) | -18.4 | 28.1 | 0.51 | 0.8 | 17.8 | 0.96 | -19.1 | 13.5 | 0.16 | -4.4 | 29.0 | 0.88 | 16.3 | 14.8 | 0.27 | 3.0 | 6.3 | 0.64 | 1.6 | 3.5 | 0.65 | 0.7 | 9.8 | 0.95 |
| Sex (ref female) | -65.1 | 34.4 | 0.06 | **-45.7** | **21.7** | **0.04** | -19.7 | 16.4 | 0.23 | **113.1** | **35.5** | **<.01** | -34.1 | 18.1 | 0.06 | 3.1 | 7.7 | 0.69 | 1.3 | 4.2 | 0.76 | **-49.3** | **11.8** | **<.01** |
| Evening (ref int) | -9.9 | 48.0 | 0.84 | -20.8 | 30.4 | 0.49 | 10.9 | 23.0 | 0.63 | -13.0 | 49.6 | 0.79 | -16.0 | 25.3 | 0.53 | 3.6 | 10.8 | 0.74 | 2.2 | 5.9 | 0.71 | **44.3** | **16.7** | **0.01** |
| Morning (ref int) | 2.0 | 27.5 | 0.94 | 9.4 | 17.4 | 0.59 | -7.6 | 13.1 | 0.56 | -21.9 | 28.3 | 0.44 | 10.2 | 14.5 | 0.48 | 9.6 | 6.2 | 0.12 | 2.5 | 3.4 | 0.47 | 0.9 | 9.4 | 0.92 |
| Overweight (ref norm) | 19.8 | 32.8 | 0.55 | 13.1 | 20.7 | 0.53 | 6.8 | 15.7 | 0.66 | -41.4 | 33.8 | 0.22 | 5.6 | 17.3 | 0.75 | 3.5 | 7.4 | 0.64 | 1.3 | 4.0 | 0.75 | 6.5 | 11.3 | 0.57 |
| Obese (ref norm) | 4.2 | 31.7 | 0.89 | -5.8 | 20.0 | 0.77 | 10.0 | 15.2 | 0.51 | 19.4 | 32.7 | 0.55 | -6.9 | 16.7 | 0.68 | -5.5 | 7.1 | 0.44 | -7.8 | 3.9 | 0.05 | -0.8 | 10.9 | 0.94 |
| Weekend (ref wkday) | **58.7** | **11.7** | **<.01** | **44.6** | **8.0** | **<.01** | **14.3** | **6.7** | **0.03** | **-105.2** | **12.8** | **<.01** | 10.7 | 7.4 | 0.15 | **14.4** | **2.8** | **<.01** | **-3.0** | **1.4** | **0.03** | -2.5 | 6.7 | 0.71 |
| Education (ref <bach) | -26.1 | 30.7 | 0.40 | -8.1 | 19.4 | 0.68 | -17.9 | 14.7 | 0.22 | **71.5** | **31.7** | **0.02** | -19.1 | 16.2 | 0.24 | -3.7 | 6.9 | 0.59 | 2.9 | 3.8 | 0.44 | **-22.1** | **10.3** | **0.03** |
| Note. beta estimate units are in minutes, and significant values (p<.05) are bolded. Models estimated the associations of clinical and demographic covariates with each dependent variable. All models were adjusted for age, sex, chronotype, BMI, weekday/weekend, education, and the linear associations of change in the outcome.  rSST=recreational sedentary screen time, other-SED=other non-rSST sedentary time, STAND=standing time, LPA=light-physical activity, MVPA=moderate-vigorous physical activity, SLEEP=sleep duration.  References for clinical and demographics parameters: Age (reference 23-44 years); Sex (reference female); Evening Chronotype (reference intermediate); Morning Chronotype (reference intermediate); Overweight (reference normal); Obese (reference normal); Weekend (reference weekday); Education (reference <bachelor’s degree). | | | | | | | | | | | | | | | | | | | | | | | | |
|  |  |  |  |  |  |  |  |  |  |  |  |  |  |  |  |  |  |  |  |  |  |  |  |  |

| **Supplemental Table 3.** Interactions between total rSST and 24-hour behaviors. | | | | | | | | | | | | | | | |
| --- | --- | --- | --- | --- | --- | --- | --- | --- | --- | --- | --- | --- | --- | --- | --- |
| Total rSST and covariate interaction associations with 24-hour behaviors | | | | | | | | | | | | | | | |
|  | Model 1a: total rSST →other-SED | | |  | Model 1b: total rSST →STAND | | |  | Model 1c: total rSST →LPA | | |  | Model 1d: total rSST →MVPA | | |
| Parameter | β | SE | *p* |  | β | SE | *p* |  | β | SE | *p* |  | β | SE | *p* |
|  |  |  |  |  |  |  |  |  |  |  |  |  |  |  |  |
| between-person |  |  |  |  |  |  |  |  |  |  |  |  |  |  |  |
| Total rSST * Age | 11.97 | 9.50 | 0.21 |  | -1.95 | 7.05 | 0.78 |  | **-8.31** | **3.05** | **0.01** |  | 0.17 | 1.73 | 0.92 |
| Total rSST * Sex | 16.84 | 20.27 | 0.41 |  | -8.35 | 15.04 | 0.58 |  | -1.76 | 6.52 | 0.79 |  | -4.71 | 3.69 | 0.20 |
| Total rSST * Evening Chronotype | 18.88 | 25.87 | 0.47 |  | -8.45 | 19.19 | 0.66 |  | -7.19 | 8.30 | 0.39 |  | 7.72 | 4.69 | 0.10 |
| Total rSST * Morning Chronotype | 11.74 | 9.67 | 0.23 |  | -10.43 | 7.17 | 0.15 |  | 1.41 | 3.10 | 0.65 |  | 1.41 | 1.75 | 0.42 |
| Total rSST * Weekend | -2.97 | 4.46 | 0.51 |  | -2.31 | 3.31 | 0.48 |  | 1.43 | 1.29 | 0.27 |  | 0.29 | 0.64 | 0.66 |
|  |  |  |  |  |  |  |  |  |  |  |  |  |  |  |  |
| within-person |  |  |  |  |  |  |  |  |  |  |  |  |  |  |  |
| Total rSST * Age | -2.89 | 4.29 | 0.50 |  | -2.67 | 3.18 | 0.40 |  | 1.36 | 1.24 | 0.27 |  | -0.28 | 0.61 | 0.65 |
| Total rSST * Sex | 4.59 | 5.33 | 0.39 |  | 3.36 | 3.95 | 0.40 |  | 2.11 | 1.54 | 0.17 |  | -0.14 | 0.76 | 0.85 |
| Total rSST * Evening Chronotype | -3.49 | 6.01 | 0.56 |  | -5.84 | 4.45 | 0.19 |  | 1.59 | 1.73 | 0.36 |  | 1.13 | 0.86 | 0.19 |
| Total rSST * Morning Chronotype | 3.61 | 4.32 | 0.40 |  | -0.04 | 3.21 | 0.99 |  | **-3.40** | **1.25** | **0.01** |  | -0.47 | 0.62 | 0.45 |
| Total rSST * Weekend | 5.53 | 4.45 | 0.21 |  | -4.06 | 3.30 | 0.22 |  | -2.27 | 1.29 | 0.08 |  | -0.38 | 0.64 | 0.55 |
|  |  |  |  |  |  |  |  |  |  |  |  |  |  |  |  |
| 24-hour behavior and covariate interaction associations with total rSST | | | | | | | | | | | | | | | |
|  | Model 2a: other-SED →total rSST | | |  | Model 2b: STAND →total rSST | | |  | Model 2c: LPA  →total rSST | | |  | Model 2d: MVPA →total rSST | | |
| Parameter | β | SE | *p* |  | β | SE | *p* |  | β | SE | *p* |  | β | SE | *p* |
|  |  |  |  |  |  |  |  |  |  |  |  |  |  |  |  |
| between-person |  |  |  |  |  |  |  |  |  |  |  |  |  |  |  |
| 24-hour behavior * Age | 5.53 | 7.68 | 0.47 |  | 1.07 | 23.03 | 0.96 |  | **-125.37** | **58.59** | **0.03** |  | 113.00 | 116.75 | 0.33 |
| 24-hour behavior * Sex | 26.02 | 13.86 | 0.06 |  | -14.05 | 31.55 | 0.66 |  | -6.26 | 68.03 | 0.93 |  | 64.72 | 125.94 | 0.61 |
| 24-hour behavior * Evening Chronotype | **36.55** | **14.68** | **0.01** |  | -78.17 | 49.45 | 0.11 |  | -119.96 | 117.10 | 0.31 |  | **364.74** | **178.33** | **0.04** |
| 24-hour behavior * Morning Chronotype | -6.85 | 7.89 | 0.39 |  | -17.59 | 22.42 | 0.43 |  | 12.71 | 56.14 | 0.82 |  | 167.71 | 107.73 | 0.12 |
| 24-hour behavior * Weekend | 6.47 | 3.69 | 0.08 |  | **-23.47** | **10.12** | **0.02** |  | -42.85 | 25.43 | 0.09 |  | 34.67 | 44.74 | 0.44 |
|  |  |  |  |  |  |  |  |  |  |  |  |  |  |  |  |
| within-person |  |  |  |  |  |  |  |  |  |  |  |  |  |  |  |
| 24-hour behavior * Age | 5.62 | 3.38 | 0.10 |  | -4.47 | 8.04 | 0.58 |  | -12.47 | 20.54 | 0.54 |  | -43.64 | 43.60 | 0.32 |
| 24-hour behavior * Sex | **8.95** | **4.05** | **0.03** |  | 6.76 | 9.44 | 0.47 |  | 38.76 | 21.04 | 0.07 |  | 0.44 | 48.01 | 0.99 |
| 24-hour behavior * Evening Chronotype | -4.42 | 4.91 | 0.37 |  | -16.16 | 12.35 | 0.19 |  | 53.99 | 39.98 | 0.18 |  | 101.42 | 88.71 | 0.25 |
| 24-hour behavior * Morning Chronotype | 0.33 | 3.41 | 0.92 |  | 7.40 | 7.91 | 0.35 |  | -15.22 | 21.05 | 0.47 |  | -21.43 | 40.97 | 0.60 |
| 24-hour behavior * Weekend | -2.79 | 3.72 | 0.45 |  | -14.76 | 8.25 | 0.07 |  | -11.00 | 22.74 | 0.63 |  | -24.12 | 44.38 | 0.59 |
|  |  |  |  |  |  |  |  |  |  |  |  |  |  |  |  |
| Note. beta estimates (β) and standard errors (SE) are scaled hourly. Significant p-values (p) are bolded (p<0.05). Data are in minutes.  Model 1 examined the interaction associations of total rSST (independent variable) with 24-hour behaviors, other-SED (model 1a), STAND (model 1b), LPA (model 1c), and MVPA (model 1d) (dependent variables), and with variables, age, sex, chronotype, and weekday/weekend.  Model 2 examined the interaction associations of 24-hour behaviors, other-SED (model 2a), STAND (model 2b), LPA (model 2c), and MVPA (model 2d), (independent variables) with total rSST (dependent variable), and with variables, age, sex, chronotype, and weekday/weekend.  All models were adjusted for age, sex, chronotype, BMI, weekday/weekend, education, and the linear associations of change in the outcome. Covariate references are age: reference 23-44 years; sex: reference female; chronotype: reference intermediate; and weekday/weekend: reference weekday.  rSST=recreational sedentary screen time, other-SED=other non-rSST sedentary time, STAND=standing time, LPA=light-physical activity, MVPA=moderate-vigorous physical activity. | | | | | | | | | | | | | | | |

| **Supplemental Table 4.** Interactions between daytime rSST and 24-hour behaviors. | | | | | | | | | | | | | | | |
| --- | --- | --- | --- | --- | --- | --- | --- | --- | --- | --- | --- | --- | --- | --- | --- |
| Daytime rSST and covariate interaction associations with 24-hour behaviors | | | | | | | | | | | | | | | |
|  | Model 1.1a: daytime rSST→other-SED | | |  | Model 1.1b: daytime rSST→STAND | | |  | Model 1.1c: daytime rSST→LPA | | |  | Model 1.1d: daytime rSST→MVPA | | |
| Parameter | β | SE | *p* |  | β | SE | *p* |  | β | SE | *p* |  | β | SE | *p* |
|  |  |  |  |  |  |  |  |  |  |  |  |  |  |  |  |
| between-person |  |  |  |  |  |  |  |  |  |  |  |  |  |  |  |
| Daytime rSST * Age | 15.74 | 16.03 | 0.33 |  | 1.35 | 10.81 | 0.90 |  | **-12.57** | **4.59** | **0.01** |  | 1.27 | 2.65 | 0.63 |
| Daytime rSST * Sex | 56.83 | 42.40 | 0.18 |  | -41.96 | 28.60 | 0.14 |  | -17.06 | 12.16 | 0.16 |  | -12.23 | 7.02 | 0.08 |
| Daytime rSST * Evening Chronotype | 49.72 | 54.61 | 0.36 |  | -21.25 | 36.83 | 0.56 |  | -17.11 | 15.67 | 0.28 |  | 7.73 | 9.04 | 0.39 |
| Daytime rSST * Morning Chronotype | 17.05 | 16.12 | 0.29 |  | -11.26 | 10.88 | 0.30 |  | 5.36 | 4.62 | 0.25 |  | 1.30 | 2.66 | 0.63 |
| Daytime rSST * Weekend | -3.03 | 7.67 | 0.69 |  | -6.61 | 5.29 | 0.21 |  | 3.70 | 2.04 | 0.07 |  | 0.41 | 1.01 | 0.69 |
|  |  |  |  |  |  |  |  |  |  |  |  |  |  |  |  |
| within-person |  |  |  |  |  |  |  |  |  |  |  |  |  |  |  |
| Daytime rSST * Age | -8.60 | 6.67 | 0.20 |  | -0.81 | 4.60 | 0.86 |  | 1.81 | 1.78 | 0.31 |  | -0.38 | 0.88 | 0.66 |
| Daytime rSST * Sex | 8.33 | 8.65 | 0.34 |  | 4.02 | 5.97 | 0.50 |  | 0.29 | 2.31 | 0.90 |  | -0.53 | 1.14 | 0.64 |
| Daytime rSST * Evening Chronotype | **-28.16** | **11.06** | **0.01** |  | -12.02 | 7.63 | 0.12 |  | 2.38 | 2.95 | 0.42 |  | 0.87 | 1.46 | 0.55 |
| Daytime rSST * Morning Chronotype | 9.15 | 6.68 | 0.17 |  | -3.93 | 4.61 | 0.39 |  | **-4.78** | **1.78** | **0.01** |  | -0.86 | 0.88 | 0.33 |
| Daytime rSST * Weekend | 5.06 | 7.28 | 0.49 |  | -8.83 | 5.02 | 0.08 |  | **-4.97** | **1.95** | **0.01** |  | -1.22 | 0.97 | 0.21 |
|  |  |  |  |  |  |  |  |  |  |  |  |  |  |  |  |
| 24-hour behavior and covariate interaction associations with daytime rSST | | | | | | | | | | | | | | | |
|  | Model 1.2a: other-SED→daytime rSST | | |  | Model 1.2b: STAND →daytime rSST | | |  | Model 1.2c: LPA →daytime rSST | | |  | Model 1.2d: MVPA →daytime rSST | | |
| Parameter | β | SE | *p* |  | β | SE | *p* |  | β | SE | *p* |  | β | SE | *p* |
|  |  |  |  |  |  |  |  |  |  |  |  |  |  |  |  |
| between-person |  |  |  |  |  |  |  |  |  |  |  |  |  |  |  |
| 24-hour behavior * Age | 0.81 | 5.12 | 0.87 |  | 3.32 | 14.74 | 0.82 |  | -72.54 | 36.91 | 0.05 |  | 87.40 | 74.50 | 0.24 |
| 24-hour behavior * Sex | **24.79** | **9.24** | **0.01** |  | -7.07 | 20.18 | 0.73 |  | -15.84 | 42.85 | 0.71 |  | 20.70 | 80.33 | 0.80 |
| 24-hour behavior * Evening Chronotype | **25.78** | **9.79** | **0.01** |  | -31.67 | 31.54 | 0.32 |  | -65.02 | 73.73 | 0.38 |  | 162.23 | 113.81 | 0.15 |
| 24-hour behavior * Morning Chronotype | -9.68 | 5.25 | 0.07 |  | -6.61 | 14.35 | 0.65 |  | 33.99 | 35.36 | 0.34 |  | 90.17 | 68.75 | 0.19 |
| 24-hour behavior * Weekend | 3.68 | 2.73 | 0.18 |  | -11.29 | 6.96 | 0.11 |  | -32.99 | 17.27 | 0.06 |  | **68.20** | **30.16** | **0.02** |
|  |  |  |  |  |  |  |  |  |  |  |  |  |  |  |  |
| within-person |  |  |  |  |  |  |  |  |  |  |  |  |  |  |  |
| 24-hour behavior * Age | 1.85 | 2.50 | 0.46 |  | 0.89 | 5.53 | 0.87 |  | -9.25 | 13.96 | 0.51 |  | -30.56 | 29.39 | 0.30 |
| 24-hour behavior * Sex | **7.99** | **2.99** | **0.01** |  | 5.33 | 6.49 | 0.41 |  | 10.78 | 14.29 | 0.45 |  | 15.11 | 32.36 | 0.64 |
| 24-hour behavior * Evening Chronotype | -0.09 | 3.63 | 0.98 |  | -6.31 | 8.49 | 0.46 |  | 22.42 | 27.17 | 0.41 |  | 7.45 | 59.80 | 0.90 |
| 24-hour behavior * Morning Chronotype | 0.11 | 2.52 | 0.96 |  | -2.58 | 5.44 | 0.64 |  | -20.65 | 14.30 | 0.15 |  | -27.92 | 27.62 | 0.31 |
| 24-hour behavior * Weekend | 2.89 | 2.74 | 0.29 |  | -9.40 | 5.66 | 0.10 |  | -15.39 | 15.40 | 0.32 |  | -35.28 | 29.87 | 0.24 |
|  |  |  |  |  |  |  |  |  |  |  |  |  |  |  |  |
| Note. beta estimates (β) and standard errors (SE) are scaled hourly. Significant p-values (p) are bolded (p<0.05). Data are in minutes. Model 1.1 examined the interaction associations of daytime rSST (independent variable) with 24-hour behaviors, other-SED (model 1.1a), STAND (model 1.1b), LPA (model 1.1c), and MVPA (model 1.1d) (dependent variables), and with variables, age, sex, chronotype, and weekday/weekend. Model 1.2 examined the interaction associations of 24-hour behaviors, other-SED (model 1.2a), STAND (model 1.2b), LPA (model 1.2c), and MVPA (model 1.2d) (independent variables) with daytime rSST (dependent variable), and with variables, age, sex, chronotype, and weekday/weekend. All models were adjusted for age, sex, chronotype, BMI, weekday/weekend, education, and the linear associations of change in the outcome. Covariate references are age: reference 23-44 years; sex: reference female; chronotype: reference intermediate; and weekday/weekend: reference weekday. rSST=recreational sedentary screen time, other-SED=other non-rSST sedentary time, STAND=standing time, LPA=light-physical activity, MVPA=moderate-vigorous physical activity. | | | | | | | | | | | | | | | |

| **Supplemental Table 5.** Interactions between evening rSST and 24-hour behaviors. | | | | | | | | | | | | | | | |
| --- | --- | --- | --- | --- | --- | --- | --- | --- | --- | --- | --- | --- | --- | --- | --- |
| Evening rSST and covariate interaction associations with 24-hour behaviors | | | | | | | | | | | | | | | |
|  | Model 2.1a: evening rSST→other-SED | | |  | Model 2.1b: evening rSST→STAND | | |  | Model 2.1c: evening rSST→LPA | | |  | Model 2.1d: evening rSST→MVPA | | |
| Parameter | β | SE | *p* |  | β | SE | *p* |  | β | SE | *p* |  | β | SE | *p* |
|  |  |  |  |  |  |  |  |  |  |  |  |  |  |  |  |
| between-person |  |  |  |  |  |  |  |  |  |  |  |  |  |  |  |
| Evening rSST * Age | 26.51 | 23.75 | 0.26 |  | -7.84 | 15.39 | 0.61 |  | -12.62 | 6.74 | 0.06 |  | -1.13 | 3.77 | 0.76 |
| Evening rSST * Sex | 40.96 | 35.04 | 0.24 |  | 10.34 | 22.70 | 0.65 |  | 6.06 | 9.95 | 0.54 |  | -3.27 | 5.57 | 0.56 |
| Evening rSST * Evening Chronotype | 9.67 | 51.16 | 0.85 |  | -33.19 | 33.14 | 0.32 |  | -17.99 | 14.50 | 0.22 |  | 11.87 | 8.10 | 0.14 |
| Evening rSST * Morning Chronotype | 19.98 | 24.65 | 0.42 |  | -29.89 | 15.97 | 0.06 |  | -4.65 | 7.00 | 0.51 |  | 3.13 | 3.91 | 0.42 |
| Evening rSST * Weekend | -12.71 | 10.78 | 0.24 |  | -0.68 | 6.94 | 0.92 |  | -0.35 | 2.70 | 0.90 |  | 0.54 | 1.33 | 0.68 |
|  |  |  |  |  |  |  |  |  |  |  |  |  |  |  |  |
| within-person |  |  |  |  |  |  |  |  |  |  |  |  |  |  |  |
| Evening rSST * Age | -8.88 | 9.15 | 0.33 |  | -10.00 | 5.89 | 0.09 |  | 0.83 | 2.29 | 0.72 |  | -0.59 | 1.13 | 0.60 |
| Evening rSST * Sex | 8.16 | 10.58 | 0.44 |  | 3.52 | 6.81 | 0.61 |  | **6.24** | **2.64** | **0.02** |  | 0.26 | 1.30 | 0.84 |
| Evening rSST * Evening Chronotype | -5.18 | 12.08 | 0.67 |  | -10.10 | 7.78 | 0.19 |  | 2.21 | 3.02 | 0.46 |  | 2.13 | 1.49 | 0.15 |
| Evening rSST * Morning Chronotype | -0.46 | 9.23 | 0.96 |  | 7.45 | 5.94 | 0.21 |  | -3.25 | 2.31 | 0.16 |  | 0.02 | 1.14 | 0.99 |
| Evening rSST * Weekend | -12.41 | 9.12 | 0.17 |  | -5.11 | 5.87 | 0.39 |  | -0.62 | 2.29 | 0.79 |  | 0.27 | 1.13 | 0.81 |
|  |  |  |  |  |  |  |  |  |  |  |  |  |  |  |  |
| 24-hour behavior and covariate interaction associations with evening rSST | | | | | | | | | | | | | | | |
|  | Model 2.2a: other-SED→evening rSST | | |  | Model 2.2b: STAND →evening rSST | | |  | Model 2.2c: LPA →evening rSST | | |  | Model 2.2d: MVPA →evening rSST | | |
| Parameter | β | SE | *p* |  | β | SE | *p* |  | β | SE | *p* |  | β | SE | *p* |
|  |  |  |  |  |  |  |  |  |  |  |  |  |  |  |  |
| between-person |  |  |  |  |  |  |  |  |  |  |  |  |  |  |  |
| 24-hour behavior * Age | 4.50 | 4.31 | 0.30 |  | -2.53 | 11.16 | 0.82 |  | -53.51 | 28.28 | 0.06 |  | 26.46 | 55.69 | 0.63 |
| 24-hour behavior * Sex | 0.82 | 7.76 | 0.92 |  | -7.41 | 15.28 | 0.63 |  | 9.01 | 32.83 | 0.78 |  | 44.97 | 59.96 | 0.45 |
| 24-hour behavior * Evening Chronotype | 10.81 | 8.23 | 0.19 |  | -46.97 | 23.77 | 0.05 |  | -56.19 | 56.45 | 0.32 |  | **205.30** | **85.07** | **0.02** |
| 24-hour behavior * Morning Chronotype | 2.89 | 4.41 | 0.51 |  | -11.21 | 10.87 | 0.30 |  | -21.66 | 27.11 | 0.42 |  | 78.69 | 51.40 | 0.13 |
| 24-hour behavior * Weekend | 2.68 | 2.45 | 0.27 |  | **-12.15** | **5.82** | **0.04** |  | -10.40 | 14.54 | 0.47 |  | -32.29 | 25.47 | 0.21 |
|  |  |  |  |  |  |  |  |  |  |  |  |  |  |  |  |
| within-person |  |  |  |  |  |  |  |  |  |  |  |  |  |  |  |
| 24-hour behavior * Age | 3.76 | 2.25 | 0.09 |  | -5.36 | 4.63 | 0.25 |  | -3.26 | 11.75 | 0.78 |  | -13.02 | 24.83 | 0.60 |
| 24-hour behavior * Sex | 0.92 | 2.69 | 0.73 |  | 1.42 | 5.43 | 0.79 |  | **28.18** | **12.04** | **0.02** |  | -14.71 | 27.34 | 0.59 |
| 24-hour behavior * Evening Chronotype | -4.26 | 3.26 | 0.19 |  | -9.85 | 7.10 | 0.17 |  | 31.17 | 22.87 | 0.17 |  | 93.76 | 50.52 | 0.06 |
| 24-hour behavior * Morning Chronotype | 0.26 | 2.26 | 0.91 |  | 9.98 | 4.55 | 0.03 |  | 5.27 | 12.04 | 0.66 |  | 6.43 | 23.33 | 0.78 |
| 24-hour behavior * Weekend | **-6.11** | **2.45** | **0.01** |  | -5.31 | 4.71 | 0.26 |  | 6.04 | 12.89 | 0.64 |  | 11.88 | 25.12 | 0.64 |
|  |  |  |  |  |  |  |  |  |  |  |  |  |  |  |  |
| Note. beta estimates (β) and standard errors (SE) are scaled hourly. Significant p-values (p) are bolded (p<0.05). Data are in minutes. Model 2.1 examined the interaction associations of evening rSST (independent variable) with 24-hour behaviors, other-SED (model 2.1a), STAND (model 2.1b), LPA (model 2.1c), and MVPA (model 2.1d) (dependent variables), and with variables, age, sex, chronotype, and weekday/weekend.  Model 2.2 examined the interaction associations of 24-hour behaviors, other-SED (model 2.2a), STAND (model 2.2b), LPA (model 2.2c), and MVPA (model 2.2d) (independent variables) with evening rSST (dependent variable), and with variables, age, sex, chronotype, and weekday/weekend.  All models were adjusted for age, sex, chronotype, BMI, weekday/weekend, education, and the linear associations of change in the outcome. Covariate references are age: reference 23-44 years; sex: reference female; chronotype: reference intermediate; and weekday/weekend: reference weekday. rSST=recreational sedentary screen time, other-SED=other non-rSST sedentary time, STAND=standing time, LPA=light-physical activity, MVPA=moderate-vigorous physical activity. | | | | | | | | | | | | | | | |

| **Supplemental Table 6.** Interactions between rSST variables and SLEEP. | | | | | | | | | | | |
| --- | --- | --- | --- | --- | --- | --- | --- | --- | --- | --- | --- |
| rSST (total, daytime, evening) variables and covariate interaction associations with subsequent night SLEEP | | | | | | | | | | | |
|  | Total rSST | | |  | Daytime rSST | | |  | Evening rSST | | |
|  | Model 3a: total rSST  →subsequent night SLEEP | | |  | Model 3b: daytime rSST  →subsequent night SLEEP | | |  | Model 3c: evening rSST  →subsequent night SLEEP | | |
| Parameter | β | SE | *p* |  | β | SE | *p* |  | β | SE | *p* |
|  |  |  |  |  |  |  |  |  |  |  |  |
| between-person |  |  |  |  |  |  |  |  |  |  |  |
| rSST variable * Age | 5.42 | 4.82 | 0.26 |  | 9.10 | 7.12 | 0.20 |  | 3.65 | 11.36 | 0.75 |
| rSST variable * Sex | 19.48 | 10.31 | 0.06 |  | 37.48 | 18.70 | 0.05 |  | 14.70 | 16.03 | 0.36 |
| rSST variable * Evening Chronotype | -4.00 | 13.19 | 0.76 |  | -18.02 | 26.67 | 0.50 |  | 12.99 | 22.32 | 0.56 |
| rSST variable * Morning Chronotype | 5.45 | 4.81 | 0.26 |  | 6.05 | 7.07 | 0.39 |  | 19.21 | 11.30 | 0.09 |
| rSST variable * Weekend | 0.96 | 3.04 | 0.75 |  | -0.12 | 4.85 | 0.98 |  | 4.89 | 6.28 | 0.44 |
|  |  |  |  |  |  |  |  |  |  |  |  |
| within-person |  |  |  |  |  |  |  |  |  |  |  |
| rSST variable * Age | 0.98 | 2.94 | 0.74 |  | 1.12 | 4.19 | 0.79 |  | 2.61 | 5.45 | 0.63 |
| rSST variable * Sex | 6.19 | 3.80 | 0.10 |  | 2.69 | 5.49 | 0.62 |  | **14.36** | **6.34** | **0.02** |
| rSST variable * Evening Chronotype | 0.12 | 5.95 | 0.98 |  | 5.37 | 8.51 | 0.53 |  | -2.54 | 9.62 | 0.79 |
| rSST variable * Morning Chronotype | -0.97 | 2.95 | 0.74 |  | -3.08 | 4.17 | 0.46 |  | 0.95 | 5.46 | 0.86 |
| rSST variable * Weekend | 1.00 | 3.04 | 0.74 |  | 3.06 | 4.48 | 0.49 |  | -0.28 | 5.43 | 0.96 |
|  |  |  |  |  |  |  |  |  |  |  |  |
| Previous night SLEEP and covariate interaction associations with rSST (total, daytime, evening) variables | | | | | | | | | | | |
|  | Total rSST | | |  | Daytime rSST | | |  | Evening rSST | | |
|  | Model 4a: previous night SLEEP→next day total rSST | | |  | Model 4b: previous night SLEEP→next day daytime rSST | | |  | Model 4c: previous night SLEEP→next day evening rSST | | |
| Parameter | β | SE | *p* |  | β | SE | *p* |  | β | SE | *p* |
|  |  |  |  |  |  |  |  |  |  |  |  |
| between-person |  |  |  |  |  |  |  |  |  |  |  |
| SLEEP * Age | 50.60 | 39.68 | 0.20 |  | 42.29 | 25.40 | 0.10 |  | 8.43 | 19.10 | 0.66 |
| SLEEP * Sex | **123.88** | **56.21** | **0.03** |  | **78.62** | **35.97** | **0.03** |  | 45.81 | 27.04 | 0.09 |
| SLEEP * Evening Chronotype | -18.38 | 66.56 | 0.78 |  | -4.30 | 42.47 | 0.92 |  | -13.82 | 31.84 | 0.66 |
| SLEEP * Morning Chronotype | 17.74 | 39.50 | 0.65 |  | 3.57 | 25.30 | 0.89 |  | 14.80 | 19.03 | 0.44 |
| SLEEP * Weekend | 7.82 | 15.25 | 0.61 |  | 5.36 | 10.57 | 0.61 |  | 2.68 | 8.49 | 0.75 |
|  |  |  |  |  |  |  |  |  |  |  |  |
| within-person |  |  |  |  |  |  |  |  |  |  |  |
| SLEEP * Age | 0.92 | 11.06 | 0.93 |  | 1.23 | 7.67 | 0.87 |  | -0.36 | 6.17 | 0.95 |
| SLEEP * Sex | -12.09 | 11.89 | 0.31 |  | -3.19 | 8.26 | 0.70 |  | -8.93 | 6.64 | 0.18 |
| SLEEP * Evening Chronotype | -18.58 | 22.38 | 0.41 |  | -8.55 | 15.53 | 0.58 |  | -10.09 | 12.49 | 0.42 |
| SLEEP * Morning Chronotype | 7.62 | 11.00 | 0.49 |  | 6.81 | 7.63 | 0.37 |  | 0.79 | 6.14 | 0.90 |
| SLEEP * Weekend | 10.63 | 12.70 | 0.40 |  | -0.12 | 8.79 | 0.99 |  | 10.30 | 7.06 | 0.15 |
|  |  |  |  |  |  |  |  |  |  |  |  |
| Note. beta estimates (β) and standard errors (SE) are scaled hourly. Significant p-values (p) are bolded (p<0.05). Data are in minutes.  Model 3 examined the interaction associations of total rSST (3a), daytime rSST(3b), and evening rSST (3c) variables (independent variable) with subsequent night SLEEP (dependent variable), and with variables, age, sex, chronotype, and weekday/weekend.  Model 4 examined the interaction associations of the previous night's SLEEP (independent variable) with the next day's total rSST (4a), daytime rSST (4b), and evening rSST (4c) variables (dependent variable), and with variables, age, sex, chronotype, and weekday/weekend.  All models were adjusted for age, sex, chronotype, BMI, weekday/weekend, education, and the linear associations of change in the outcome. Covariate references are age: reference 23-44 years; sex: reference female; chronotype: reference intermediate; and weekday/weekend: reference weekday. rSST=recreational sedentary screen time, SLEEP=sleep duration. | | | | | | | | | | | |
